# The HARDSHIP databases: a forthcoming free good from the Global Campaign against Headache

**DOI:** 10.1186/s10194-023-01554-9

**Published:** 2023-03-06

**Authors:** Timothy J. Steiner, Andreas Husøy, Hallie Thomas, Lars Jacob Stovner

**Affiliations:** 1 Department of Neuromedicine and Movement Science, NorHEAD, Norwegian University of Science and Technology, Edvard Griegs Gate, Trondheim, Norway; 2 Department of Neurology, University of Copenhagen, Copenhagen, Denmark; 3 Division of Brain Sciences, Imperial College London, London, UK; 4 Department of Neurology and Clinical Neurophysiology, Norwegian Advisory Unit On Headaches, St Olavs Hospital, Trondheim, Norway

**Keywords:** HARDSHIP questionnaire, Headache, Prevalence, Burden, Big data, Health policy, Global Campaign against Headache

## Abstract

In order to pursue its purpose of reducing the global burden of headache, the Global Campaign against Headache has gathered data on headache-attributed burden from countries worldwide. These data, from the individual participants in adult population-based studies and child and adolescent schools-based studies, are being collated in two databases, which will be powerful resources for research and teaching and rich information sources for health policy.

Here we briefly describe the structure and content of these databases, and announce the intention to make them available in due course as a free good.

## Background

The Global Campaign against Headache has been active for nearly two decades, with three strategic objectives [1]. Its ultimate objective, *Action for change*, required the development of evidence-based recommendations for intervention, justified by cost-effective analysis. Necessarily preceding this was *Awareness for action*: agitating for change by promoting awareness of the need for change among the agents for change (principally politicians and health-care providers, but also employers, schools and the general public). The foundation for this was *Knowledge*: knowledge to establish – and demonstrate – what it was that required change, and why [1]. Primary headache disorders are largely remediable causes of public ill health and disability, and of high financial cost, but 20 years ago these consequences were little recognised and poorly quantified.

So began the Campaign’s series of population-based studies to gather evidence, initially among adults (aged 18–65 years), of the scope and scale of the global burden of headache, and its collation of this evidence [1]. Schools-based studies among children (aged 6–11 years) and adolescents (aged 12–17 years) commenced later [1].

The adult studies have used standardised methodology [2] and the Headache-Attributed Restriction, Disability and Impaired Participation (HARDSHIP) structured questionnaire [3], both developed by an international expert consensus group. This programme is nearing completion, with national or sub-national studies conducted in all world regions: African (Ethiopia [4, 5], Zambia [6, 7], and Benin, Cameroon and Mali [not yet published]); American (Peru [not yet published]); Eastern Mediterranean (Pakistan [8, 9], Saudi Arabia [10] and Morocco [not yet published]); European (Lithuania [11], Russia [12–15] and, within the Eurolight project, eight other countries of western Europe [16–21]); South East Asia (India south [Karnataka State] [22–26], India north [Delhi and National Capital Territory Region] [not yet published] and Nepal [27–36]); and Western Pacific (China [37–42] and Mongolia [43]).

The child and adolescent studies use different (schools-based) but also standardised methodology, and cut-down versions of the HARDSHIP questionnaire [44]. This global programme, interrupted by the SARS-CoV-2 pandemic, is again ongoing. Studies have been completed in Austria [45], Ethiopia [46], Iran [47], Lithuania [48, 49], Mongolia [50], Turkey [51] and Zambia [52], and in Benin, Nepal and Serbia [not yet published]. Others have commenced or are planned.

All of these studies were, or are being, conducted with ethics approvals for use of the anonymised data to expand knowledge and inform policy, creating a potentially powerful resource. The individual-participant data (IPD) collectively offer a very broad understanding of headache-attributed burden, the full spectrum of which goes far beyond symptom burden and disability, encompassing burdens outside the attack, including those that are cumulative over a lifetime, and burdens on others than those immediately affected: family, friends and colleagues of those with headache, and society [3].

The Global Campaign is committed to making these data freely available, as a resource for research and teaching and as an information source for policy.

## The HARDSHIP databases

Both under construction, the databases will capture all IPD from these studies, with sub-datasets describing sampling and other methodology as attributes of the main datasets.

### The adult database

This database, structured in line with the adult HARDSHIP questionnaire [3], is made up of multiple modules. Each module covers a particular domain, with one or more sub-modules, most of which have multiple fields. In some modules, previously validated instruments are embedded.

In the first domain, completed post-survey, are study characteristics, including quality evaluation. The first enquiry domain captures demographic and social IPD. The headache module includes the screening questions and, when present, characterisation of the most bothersome headache (MBH). Within the burden module are symptom burden, lost productive time (questions from the Headache-Attributed Lost Time [HALT] indices [53]), interictal burden, impacts on educational attainment, income, children, partners and colleagues, social life, love life and family planning, and perceptions of control, quality of life (QoL: questions from WHOQoL-8 [54]), and wellbeing (four questions used by the UK Office of National Statistics in the national census [55]). A sub-module relates to headache yesterday (*ie*, headache on the day preceding the survey), with IPD regarding headache characteristics and lost time that are largely free from recall error. The health-care utilisation module covers acute and preventative medication, professional care, and investigations conducted for diagnostic or management purposes. The comorbidities module captures weight, height, waist circumference and blood pressure, and includes the Hospital Anxiety and Depression Scale (HADS) [56], the Shona Symptom Questionnaire [57] and the neuroticism subscale of the Eysenck Personality Questionnaire [30].

All contributing adult studies used the adult HARDSHIP questionnaire [3], but the modular structure of this questionnaire allowed selective inclusion of modules according to study purpose and/or the resources available. The demographic and headache modules, and some measures of burden, were always necessary.

### The child and adolescent database

The child and adolescent HARDSHIP questionnaires are much reduced versions of the adult questionnaire, recognising the limitations of enquiry among young people, and the time constraints imposed by conducting the enquiry in class [45]. The headache module is nevertheless very similar. The burden module is focused on impact on education (lost schooldays), but also covers other (out of school) activities, which are important to these age groups. A sub-module captures time lost by parents from work while tending to their son’s or daughter’s headache. Other burden sub-modules use elements from PedMIDAS [58], and selected (headache-relevant) questions from KINDL® [59] to address concentration, emotional impact and QoL [45].

### Diagnostic module

Each database has a final module, also completed post-survey, containing algorithmically derived diagnoses from the characterisation of MBH according to ICHD criteria [60], as far as these can be applied to cross-sectional enquiries [2]. The principal limitations here are two-fold. The first relates to the final criterion in ICHD for all primary headache disorders: “Not better explained by another ICHD diagnosis” [60]. Epidemiological enquiry (as opposed to clinical) cannot exclude all other possible causes [2]. The second relates to diagnosis of headache reported on ≥ 15 days/month, which may include chronic migraine, chronic tension-type headache and medication-overuse headache (MOH), trigeminal autonomic cephalalgias (although these have very low probability of occurrence in samples typically of N ~ 2,000 [61]), new daily-persistent headache (also rare) and, potentially, any of a small range of other, relatively uncommon, secondary headache disorders [60]. These can be identified only by expert questioning, usually with follow-up [62]. For a diagnosis of probable MOH (pMOH), the module includes frequency of use of acute medication as an association, without evidence of causation [60, 63].

For adults, the diagnostic possibilities include pMOH, other headache on ≥ 15 days/month, definite migraine, definite tension-type headache (TTH), probable migraine and probable TTH. This strict order observes the diagnostic hierarchy of ICHD [60]. Remaining cases are unclassified. It has been outside the scope of the contributing studies, and is beyond the ability of HARDSHIP [3], to detect secondary headaches other than pMOH. These, if not among other headache on  ≥ 15 days/month, are likely to fall within unclassified headache.

For children and adolescents, the same diagnostic possibilities exist, in the same order, but migraine and TTH are preceded within the algorithm by undifferentiated headache (UdH), defined as mild headache of <1 hour’s duration [51].

### Quality control

Stringent quality controls are being applied in database construction, not only to the input of datasets but also to inspection of these, prior to their inclusion, for consistency and plausibility.

Always in mind is potential fraud, with scrutiny of datasets alert to the possibilities of both invention and duplication of records. Fraud of either type having the purpose of inflating N may be perpetrated during collection of data or later, when data are first input to create datasets. Fraud during data collection is to some extent preventable, or detectable should it occur, by quality controls applied at the time [2, 64]. These controls have been written into Global Campaign protocols [2].

### Hosting and access

The HARDSHIP databases will be hosted by NorHEAD at Norwegian University of Science and Technology (NTNU). The adult database is expected to be available earlier than the child and adolescent database.

It is not yet established how hosting will be managed, balancing open access against appropriate security controls. These details will be announced in due course, along with more complete descriptions of the databases.

### Potential value

With contributory data expected from over 30 countries, the databases will, together, eventually include >80,000 IPD records. Although the number pertaining to a headache disorder will be somewhat smaller, these records will constitute a direct and detailed account of the global burden of headache across the ages 6–65 years.

The Global Burden of Disease (GBD) study, using Global Campaign and all other available data, already reports migraine as the second highest cause worldwide of years lived with disability, with TTH an additional but lesser contributor [65, 66]. GBD attaches a meaning to “disability” that might better be referred to as lost health [67–69], but offers no sense of what this means in relation to any disease. The HARDSHIP databases, based on broad enquiries into the scope of headache-attributed burden, will offer clear and unmatched insight into the range of impairments that are reflected in these assessments of lost health, and, perhaps, enable the features of headache to be identified that are most contributory to it. They will go beyond this, highlighting what is lacking in GBD’s assessments of headache-attributed burden, focused solely, as they are, on the symptomatic state [65].

Because all contributory studies to each database will have used the same protocol and questionnaire, between-country and between-region comparisons will be readily possible. These may determine whether there are universal truths in how headache affects peoples’ lives, or whether there are real variations dependent upon genetics, environment and/or culture. Association analyses, for example between headache features and various measures of health loss or other burden, will be strongly supported.

The Global Campaign has a policy of making all of its products freely available. Its clear commitment to do so with these databases will enable these values to be realised.

## Conclusion

These databases, providing a detailed account of the global burden of headache across the ages 6–65 years, will be new, powerful and unmatched resources for research and teaching, and rich information sources for health policy.

## Data Availability

Not applicable.

## Abbreviations

GBD: Global Burden of Disease (study)
HADS: Hospital Anxiety and Depression Scale
HALT: Headache-Attributed Lost Time (indices)
HARDSHIP: Headache-Attributed Restriction, Disability and Impaired Participation (structured questionnaire)
ICHD: International Classification of Headache Disorders
IPD: Individual participant data
LTB: *Lifting The Burden*
MBH: Most bothersome headache
MOH: Medication-overuse headache
NTNU: Norwegian University of Science and Technology
pMOH: Probable medication-overuse headache
QoL: Quality of life
TTH: Tension-type headache
WHOQoL: World Health Organization Quality of Life (questionnaire)
